# The Importance of Oxidative Stress and Antioxidant Metabolism for the Development of Chilling Injury in Kiwifruit

**DOI:** 10.3390/antiox15010030

**Published:** 2025-12-24

**Authors:** Chelsea Kerr, David J. Burritt, Jeremy N. Burdon

**Affiliations:** 1 Department of Botany, University of Otago, Dunedin 9016, New Zealand; 2 The New Zealand Institute for Plant and Food Research Limited, Mt. Albert, Auckland 1025, New Zealand; jeremy.burdon@plantandfood.co.nz

**Keywords:** postharvest storage, antioxidant metabolism, reactive oxygen species, chilling injury, kiwifruit

## Abstract

Fruit losses can occur during the storage of kiwifruit due to disorders such as chilling injury. A major factor influencing storage life is antioxidant metabolism. Antioxidants detoxify reactive oxygen species (ROS) and limit oxidative stress, which otherwise damages proteins, lipids, and DNA. Oxidative stress accelerates degradation and contributes to physiological disorders such as chilling injury during storage of kiwifruit. Regulation of antioxidant metabolism is complex, involving several biochemical pathways critical for maintaining kiwifruit integrity postharvest. The objective of this review is to critically evaluate current knowledge regarding oxidative stress and antioxidant metabolism and the development of postharvest disorders of kiwifruit during cold storage, with an emphasis on chilling injury. The review will provide an overview of current knowledge regarding oxidative stress and antioxidant metabolism in kiwifruit during cold storage, identifying gaps in our knowledge. The review will also discuss how an understanding of antioxidant metabolism can be used to design treatments that have the potential to increase the storability of kiwifruit and reduce chilling injury.

## 1. An Introduction to Kiwifruit in New Zealand

Kiwifruit plants are dioecious deciduous vines belonging to the genus *Actinidia* which contains over 60 species [1]. The current names for the two most common commercial cultivars in New Zealand are *Actinidia chinensis* var. *deliciosa* (commonly referred to as ‘Hayward’) and *Actinidia chinensis* var. chinensis (commonly referred to as ‘Zesy002’ and commercialized as Zespri^TM^ SunGold,). However, early papers may refer to these as *Actinidia deliciosa* and *Actinidia chinensis*. Originating from southern China, the fruit were originally called Chinese gooseberry before their commercialization in New Zealand. Export from New Zealand started in the 1960s, and by 1975 was limited to the ‘Hayward’ cultivar [2,3]. Unlike many commercial crops that have been domesticated over long periods of time, commercial kiwifruit are only one or two generations removed from their wild relatives [4]. The kiwifruit industry in New Zealand generated export returns of nearly NZD 2.7 billion in the 2022 and is therefore very valuable to New Zealand’s economy [5]. The success of the New Zealand industry was initially attributed to the favourable characteristics of ‘Hayward’ fruit, including its storage potential, enabling long term storage and shipment over long distances [2,6,7,8]. In New Zealand, kiwifruit are harvested over approximately 2–3 months (typically March–June), ‘Hayward’ being harvested later than ‘Zesy002’ and stored in cold storage for approximately 4–6 months to spread availability in the market [4].

## 2. An Overview of the Postharvest Storage of Kiwifruit

Kiwifruit, like most other fresh produce, remains metabolically active after harvest. High metabolic activity, and therefore high rates of respiration, is correlated with low postharvest potential for storage and reduced shelf life [9,10,11]. The primary goal of postharvest storage is to maintain desirable qualities (both physical and nutritional quality) of produce and to deliver the produce to market with minimal losses which can occur due to the development of postharvest disorders. Postharvest disorders are generally caused by physical, pathological or physiological mechanisms [10]. All fresh produce will inevitably deteriorate postharvest, although the rate of deterioration can be slowed by harvesting at the optimal physiological maturity and then holding in the optimal postharvest storage conditions. Both the physiological maturity and the storage conditions will depend upon the marketing plan for the produce, i.e., immediately or after short or long periods in storage. Reducing the temperature reduces the fruit’s metabolic rate and microbial growth, allowing the produce to be stored to extend marketing, or for transport to more distant markets. However, conditions which are too cold, or too long at a cold temperature, can result in physiological disorders generally termed chilling injury [12,13]. The major physiological disorders during commercial storage of *Actinidia chinensis* (kiwifruit) cultivars include, chilling injury, skin markings and shrivel [14]. The focus of this review will be the physiological disorder chilling injury during cold storage of kiwifruit.

Kiwifruit are generally considered to have a long storage life when stored at temperatures close to 0 °C [14] fruit that store well are largely dependent on the maturity of the fruit and low temperature storage [15]. Variation in the significance of postharvest disorders occurs between grower lines, season, and cultivar [16]. In New Zealand, kiwifruit growers are also encouraged to harvest fruit early in the season; kiwifruit that are harvested early in the season are known as KiwiStart fruit. These fruit are harvested at earlier maturity, and therefore a smaller size, than the later harvested MainPack fruit. Growers are offered an incentive (compensation for a reduced size fruit) to harvest early so that kiwifruit are available to the market earlier than kiwifruit from competitors [15]. The external physical characteristics of kiwifruit do not change dramatically during ripening and current harvest indices do not always reflect the physiological state of the fruit; therefore, fruit susceptible to postharvest disorders such as chilling injury can be commonly harvested [15].

Kiwifruit are usually harvested when firm and unripe; they then ripen either slowly during storage at low temperatures or more rapidly at higher temperatures, which can also be accelerated with ethylene [14]. Physiological changes involved in maturation and/or early stages of ripening that are initiated before harvest continue during storage [15]. The developmental stage of kiwifruit when harvested is important for subsequent postharvest performance, including susceptibility to the physiological disorder of chilling injury during cold storage [9,10,17]. Kiwifruit maturity or harvest indices are parameters used to indicate when the fruit is suitable to be harvested for a specific marketing purpose [15]. It is important to note the difference between physiological maturity and harvest indices. These are two separate concepts in terms of commercial applications. Physiological maturity can be viewed as the phase where the fruit has developed the capacity to ripen when removed from the parent plant and involves a wide range of biochemical processes [18]. The extremes of marketing are fruit for immediate sale—in which case some form of ripening programme may be needed [14], as these fruit will be susceptible to developing postharvest disorders such as chilling injury if stored for too long—or fruit for long-term storage, in which case ripening will occur slowly in storage. This segregation of harvest by marketing requirement has led to a significant increase in the harvest window for kiwifruit [5] and therefore an increase in the numbers of fruit that develop postharvest disorders such as chilling injury. Therefore, understanding the biological processes that lead to the development of postharvest disorders is critical to reducing the incidences of lost produce through incorrect postharvest storage procedures [19].

An increase in reactive oxygen species (ROS) and/or a reduced capacity to detoxify ROS has been shown to be involved in the development of postharvest disorders such as chilling injury in kiwifruit and many other fruit species [20,21]. However, to the best of our knowledge, no synthesis of the published information of the importance of ROS and ROS detoxification in kiwifruit is available; as such, in the following sections of this review, we aim to define the current state of knowledge surrounding antioxidant metabolism in kiwifruit, referring to other fruit species if information on kiwifruit is lacking.

## 3. Oxidative Stress and Antioxidant Metabolism and Their Importance for Fruit During Postharvest Storage

Reactive oxygen species (ROS) are unavoidable by-products of aerobic metabolism and are produced within the mitochondria, chloroplasts/plastids and peroxisomes [22]. The production of ROS is a common downstream response/consequence of exposure of plants to stress [23]. At controlled levels, ROS function as signalling molecules, regulating processes such as cell expansion, ripening, and senescence [24]. However, at high concentrations ROS can cause irreversible damage to proteins, lipids and DNA. Prolonged elevated levels of ROS, due to excessive ROS production, insufficient antioxidant enzyme activity or damage to the antioxidant system, can result in oxidative stress. Oxidative stress leads to the accumulation of damaged macromolecules, causing loss of cellular integrity and functionality, leading to physiological disorders and ultimately causing cell death [22,23,25,26]. Fruit development and ripening can be considered an oxidative process whereby ROS are produced [26,27]. Abiotic and biotic stresses such as extreme temperatures, drought, and pathogen invasion can disrupt the balance between ROS production and scavenging (removal), leading to oxidative stress and subsequent cell damage [28]. Oxidative stress and subsequent damage are evident in tissues which are sensitive to chilling temperatures [29]. One of the early physiological responses to low temperatures and chilling injury is an increase in ROS production [30,31]. Increases in ROS decrease storage life and shelf-life potential by increasing the rate of degradation and development of physiological disorders [31]. The severity and symptoms of oxidative stress depend on the site of ROS production, as well as the intensity and duration of the stress [32]. Many treatments that reduce the incidence of postharvest disorders such as chilling injuries are associated with increased antioxidant enzyme activity [20,33,34].

**Figure 1:**
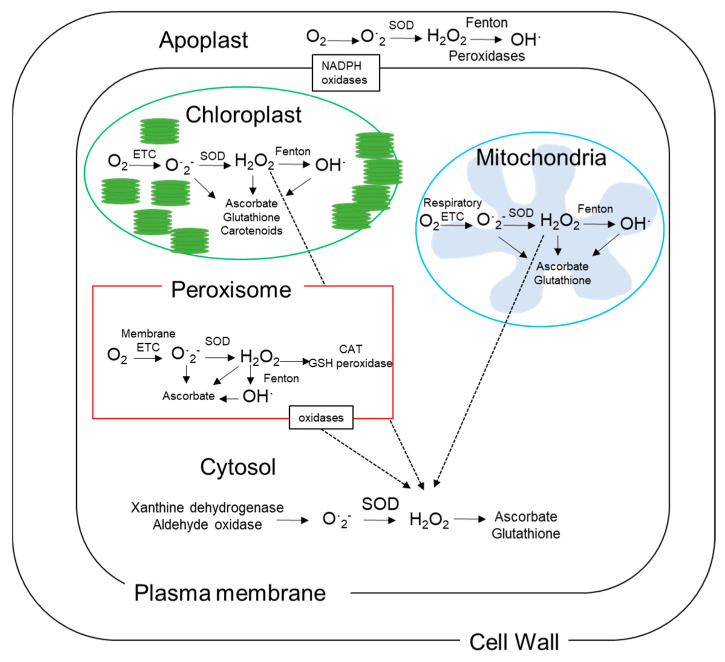
Mechanisms of reactive oxygen species (ROS) production and sites of production in plant cells. ETC refers to electrons that are transported across the electron transport chain. Superoxide dismutase (SOD). Catalase (CAT). Glutathione (GSH). Dashed arrows indicate sources of cytosolic H_2_O_2_ from chloroplast/plastids, mitochondria and/or peroxisomes.

Reactive oxygen species include superoxide (O^·^_2_^−^), hydroxyl radical (OH^−^), singlet oxygen (^1^O_2_), nitric oxide and peroxynitrite. Although hydrogen peroxide (H_2_O_2_) is not technically classified as a ROS, it is often referred to as one in the literature due to its involvement in oxidative stress processes. Ground-state triplet molecular oxygen (O_2_) is typically stable, with two parallel electrons occupying separate orbitals that rarely react with organic molecules, which typically have paired electrons with opposite spins. However, energy transfer or electron transfer processes (such as the electron transport chains in mitochondria and chloroplasts) can lead to the formation of ROS. Reactive oxygen species are naturally produced at low levels in cells during normal aerobic metabolism and photosynthesis; however, their production increases in response to stress or developmental signals which include temperature extremes, ripening, senescence, and injury [35,36]. Elevated ROS production, or insufficient scavenging capacity, can lead to oxidative damage and trigger processes including senescence if not effectively detoxified by cellular antioxidants [35,36]. Oxidases and peroxidases (such as polyamine oxidases) can also produce ROS during responses to stress such as changes in temperature [22,37,38]. The reactions which produce ROS are depicted in Table 1 and their locations in the cell where they are most commonly produced are depicted in Figure 1.

Superoxide is produced from a one-electron reduction of O_2_ but usually rapidly converted to H_2_O_2_ by the enzyme superoxide dismutase (SOD). Accumulation of superoxide can oxidize iron–sulfur clusters in proteins, leading to the irreversible formation of protein carbonyls (PC) and loss of protein function [39]. Removal of PC and resynthesis of proteins require energy and are therefore costly [40]. In the presence of ferrous ions, H_2_O_2_ can produce the highly reactive hydroxyl radical (OH^−^) via the Fenton reaction [39]. Hydroxyl radicals can also form through the interaction of H_2_O_2_ and superoxide (Haber–Weiss reaction) and are highly damaging to proteins, lipids, and DNA [39]. Oxidation of lipids results in the formation of lipid peroxides. The Haber–Weiss reaction initiates lipid peroxidation by producing OH^−^ from the reaction of H_2_O_2_ and superoxide. Lipid peroxides can exacerbate oxidative stress by generating lipid radicals, triggering a chain reaction of lipid peroxidation that requires termination by cellular antioxidants. While lipids are more susceptible to damage than DNA, DNA damage can lead to mutations that further harm cellular function [35,41,42]. Membranes play a vital role in the cell, providing barriers and compartmentalization of physiological processes, which provides a way of regulating and controlling metabolic processes. The composition of the lipid matrix in membranes is important for the function of that cell; small changes in lipid structure affecting less than 5% of the total area of the membrane can alter the function of the membrane and alter the metabolic balance in the cell. Changes in membrane lipid structures include modifications of the proportion of phospholipid polar head groups, increased desaturation of glycerolipid fatty acids, changes in the amount, composition and conjugation of membrane sterols, and changes in the amount and composition of glucocerebrosides. The development of chilling injury during postharvest storage of fruits involves a progressive increase in membrane permeability and electrolyte leakage across cell membranes beyond what normally occurs during ripening [43]. Electrolyte leakage may occur only once the fruit has been removed from cold storage and placed under ambient conditions. The extent of ion leakage has commonly been used to quantify the severity of chilling injury in fruit [43,44].

Antioxidant systems, including enzymes and organic compounds, are present in cells to detoxify ROS and to modulate ROS-based signalling. Antioxidant enzymes include SOD, catalase (CAT), glutathione peroxidase (GPOX), ascorbate peroxidase (APOX), dehydroascorbate reductase (DHAR), monodehydroascorbate reductase (MDHAR), glutathione reductase (GR) and guaiacol peroxidase (GOPX). Antioxidant compounds include ascorbate, glutathione, proline, α-tocopherols, carotenoids, flavonoids, and some alkaloids and non-proteinogenic amino acids [22,23]. Upregulation of the antioxidant systems can occur in response to stressful conditions such as high or low temperatures or significant light [32,45].

**Figure 2:**
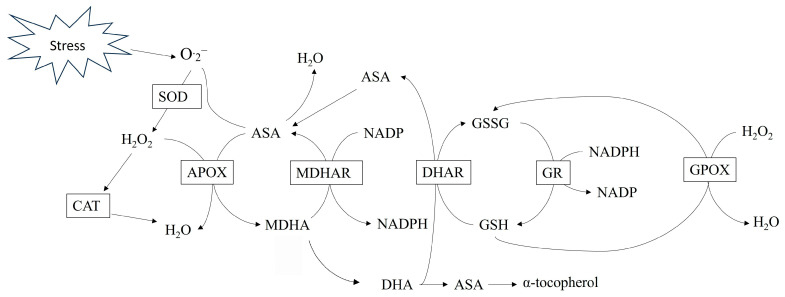
Antioxidant systems in plants. Asada–Halliwell–Foyer cycle. Superoxide anion (O·_2_^−^), superoxide dismutase (SOD), hydrogen peroxide (H_2_O_2_) catalase (CAT), ascorbate peroxidase (APOX), Ascorbate (ASA), monodehydroascorbate reductase (MDHAR), dehydroascorbic acid (DHA), dehydroascorbate reductase (DHAR), glutathione disulphide (GSSG), glutathione (GSH), glutathione reductase (GR), and glutathione peroxidase (GPOX).

Superoxide dismutase (SOD) is considered the first line of defence against ROS and is found in all organelles. By converting O^·^_2_^−^ to H_2_O_2_, the rate of formation of OH^−^ through the Haber–Weiss reaction is reduced [22]. Catalase (CAT) detoxifies H_2_O_2_ into H_2_O and O_2_. Ascorbate peroxidase (APOX) and GPOX detoxify H_2_O_2_ into O_2_. The Asada–Halliwell–Foyer cycle (Figure 2) converts H_2_O_2_ into H_2_O and recycles glutathione and ascorbate once they have been oxidized by ROS [46]. The conversion of H_2_O_2_ to H_2_O by APOX requires ascorbate as an electron donor, producing monodehydroascorbate (MDHA). Ascorbate can oxidize H_2_O_2_, forming monodehydroascorbate, which can then be reduced back to ascorbate. Monodehydroascorbate reductase (MDHAR) uses NADPH to recycle MDHA to ascorbate. Additionally, glutathione not only reacts directly with OH^−^ and ^1^O_2_ and protects the thiol groups of enzymes but also recycles oxidized ascorbate. During this process, glutathione is oxidized to glutathione disulfide (GSSG) while recycling ascorbate from dehydroascorbate via DHAR. Glutathione disulfide (GSSG) is then recycled back to glutathione by GR, which also requires NADPH [24,28,45].

Sources of ROS in fruit include the mitochondria, peroxisomes and the apoplast [47]. However, although fruit tissues have reduced photosynthetic activity compared with leaves [17], plastids are still present and are metabolically active in fruit tissues and so still contribute to the production of ROS [48]. The relative contribution of each pathway/organelle to ROS levels in kiwifruit during cold storage and the development of chilling injury has not been investigated in depth. In addition, because it is the balance between the production of ROS and the detoxification of ROS, through multiple chemical reactions and pathways, that determines the extent of oxidative stress, it is not yet possible to speculate what concentrations of ROS result in oxidative stress in kiwifruit.

## 4. Kiwifruit Ripening vs. Senescence and the Importance of Antioxidant Metabolism

At controlled levels, ROS function as signalling molecules, regulating processes such as cell expansion, ripening, and senescence [24,38]. Fruit ripening is a complex developmental process, being both genetically programmed but also responding to environmental cues. The process of fruit ripening can be considered an oxidative process; ROS are produced at controlled levels and are short-lived [26,27,38,49].

After maturation, kiwifruit pass through a discrete ripening process, most easily recognized by the rapid increase in the rate of softening and decrease in starch content [18]. While much of the change with ripening appears as in other fruit, including softening and starch breakdown, there are some specific differences in kiwifruit. First, while often referred to as being a climacteric fruit, it has long been recognized that kiwifruit (or at least the common commercial cultivars) are atypical [18,50], with most of the softening occurring prior to any increase in respiration or ethylene production. Ethylene production only increases when close to eating-soft [51] or in firmer fruit, in response to some form of damage [52]. Natural ethylene production has thus been suggested to be associated with senescence rather than ripening [18] and has been associated with ester production [53]. The role of ethylene and its effect on chilling injury is complex and is discussed in [30] and is beyond the scope of this review.

Senescence is the last stage of fruit development and ripening and involves the breakdown of cell structures and a loss of cell integrity and eventually cell death [54]. Some integrity is maintained until the final terminal phase of the senescence process to sustain enough energy to keep senescence a controlled and orderly process. However, a gradual decline in cellular regulation and function leads to a loss of membrane integrity and ultimately results in the loss of cellular homeostasis and cell death. As with other fruit, in kiwifruit the production of ROS is a normal part of senescence [55,56]. Early stages of senescence in kiwifruit may show an increase in ROS and cellular degradation processes [57], such as elevated lipoxygenase activity leading to membrane breakdown [49,58]. However, in the later stages, these activities may decrease as cells become more damaged [38].

## 5. Kiwifruit Chilling Injury and the Role of Antioxidant Metabolism

Chilling injury is a broad term used to describe disorder symptoms created by exposure to low but non-freezing temperatures, most commonly during refrigerated storage [59]. Chilling injury development involves the disruption of a large range of metabolic pathways; the exact mechanisms are unknown, but proposed mechanisms include physical changes in proteins, e.g., enzymes, increases or decreases in enzyme activity [60], phase changes in critical membrane lipid domains [59], unbalancing of critical components in metabolic pathways [60], altered metabolism and accumulation of toxins or depletion of substrates [61] and changes in cytosolic concentrations of calcium [62]. Chilling injury limits the coldest temperature that a fruit can be stored at, as well as the time it can be at that temperature, thereby limiting the potential to increase storage life through refrigeration [31]. What is too cold, depends on the specific produce, in terms of both species and the maturity at harvest. For tropical crops, temperatures below 12 °C may be damaging [63], whereas for some crops, such as kiwifruit, temperatures close to 0 °C are required before chilling injury is observed [14,64,65]. However, these temperatures vary between species, between cultivars of the same species, and depend on maturity for a single species [12,13,66].

Chilling injuries are traditionally described as a function of time by temperature, whereby symptoms appear more quickly the lower the temperature below the threshold for that fruit. Alternatively, the longer at sub-threshold temperatures, the more severe the symptoms. Chilling injury is often reviewed in this context (e.g., [13]), although there is evidence that suggests that it does not fit in all circumstances, i.e., in fruit where symptoms develop later at lower temperatures, which is usually dependent on aspects of fruit ripening, e.g., peach [67] and kiwifruit [64,68]. In these instances, these fruit flesh chilling symptoms may be regarded as a disruption of the normal orderly ripening process. In kiwifruit, the susceptibility to chilling damage is strongly maturity-dependent, with susceptibility being lost over about 6 weeks at the end of maturation and the start of ripening on the vine [69].

Chilling injury in *A. chinensis* var. *deliciosa* ‘Hayward’ kiwifruit was termed low-temperature breakdown (LTB) as a disorder resulting from long storage at low temperatures [64]. Low temperature breakdown manifests from the stylar end of the fruit, initially with a granular appearance in patches just under the skin which may progress into a continuous ring. As the disorder progresses further, the granular appearance extends towards the stem region. A water-soaked appearance of the pericarp regions can also be present, and the fruit can become very soft. In *A. chinensis* var. *chinensis* ‘Hort16A’ and ‘Zesy002’ kiwifruit, fruit with severe chilling injury may also have a dark discolouration of the skin at the stylar end [14]. Chilling injury can be a major problem in kiwifruit, mainly where early harvested fruit are over-stored. Reducing the temperature kiwifruit are stored in results in longer storage potential, although as storage temperatures decrease, the freezing point of the tissues within the fruit is approached, especially in kiwifruit which can be stored at temperatures as low as −0.5 °C [2].

The maturity of fruit has been shown to correlate with sensitivity to chilling injury; however, the concept of maturity must be defined accurately in the context of investigating the causes of chilling injury. As mentioned, “maturity” is usually defined by harvest indices, which provide an easy-to-measure snapshot of the current state of the kiwifruit in terms of starch and sugar contents, colour and firmness. They are important indicators for growers as they indicate that the fruit are competent to ripen properly postharvest. However, harvest indices do not always define the “maturity” (developmental/physiological state) of a kiwifruit well and therefore may not be ideal as indicators of susceptibility to chilling injury [15]. This is an important consideration because as kiwifruit mature, they become more tolerant to chilling injury and this has been shown to be associated with increased antioxidant defences [70].

In general, fruit that stores well for extended periods in cold storage typically produces lower levels of ROS compared to chilling sensitive fruit and/or has sufficient systems to keep ROS levels controlled [17]. Mild stress can induce beneficial adaptation without harm, while prolonged or intense stress can cause damage and cell death. Mild stress can induce adaptive metabolic and physiological responses, but prolonged or severe stress overwhelms these systems, causing damage and cell death. When multiple stresses occur simultaneously or sequentially, the fruit’s capacity to manage oxidative stress is further reduced [71,72]. Fruit that can avoid oxidative stress have a higher tolerance to chilling-induced injuries [70,72,73,74]. Different individual fruit vary in their tolerances to stressors based on the fruit species, stressor type, and their initial condition. Cold storage prolongs the postharvest life of fruits by decreasing metabolic activity and slowing the decay processes [43]. However, it can also induce a stress response, and some fruit manage cold stress better than others [32]. An early physiological response to chilling injury is an increase in the production of ROS [33,70]. At the onset of stress, plants undergo physiological changes, with cells sensing and responding to stress signals. They then activate mechanisms to adapt and repair damage. If the stress exceeds the plant’s tolerance, it leads to damage, early senescence, and eventually cell death [75,76]. Fruits manage stress at low levels by activating downstream pathways through hormone signaling, which triggers secondary messengers including calcium and low levels of ROS. These “messengers” then activate protein kinases and phosphatases, leading to the transcription of stress-responsive genes and the regulation of enzymes and metabolism such as the upregulation of antioxidant enzymes [73,77,78].

Increases in oxidative stress can alter metabolism, leading to changes in enzyme activity due to variations in enzyme turnover rates and substrate availability, potentially affecting cell wall remodeling enzymes. For example, oxidative stress can upregulate enzymes like polygalacturonase and pectin methylesterase, which are involved in cell wall degradation [79,80]. Oxidation of cell wall and cell membrane structures can also lead to changes to their structures and result in changes in fruit texture. It has been shown that treatments with antioxidants such as ascorbic acid and glutathione can reduce chilling injury incidence by scavenging ROS and therefore reduce the uncontrolled breakdown of cell wall structures that occurs during chilling injury compared to the controlled breakdown that occurs during ripening [81,82]. Therefore, managing ROS levels through antioxidant treatments could possibly be one mechanism to mitigate chilling injury symptoms in kiwifruit.

## 6. Mitigation of Oxidative Damage During Cold Storage of Kiwifruit

Many studies have shown that pre-cold storage treatments, both thermal and chemical, that alleviate chilling injury symptoms in kiwifruit have biochemical pathways associated with oxidative metabolism. Key published studies are shown in Table 2. Chilling injury studies rely on early harvested kiwifruit, as chilling injury occurs at low frequencies in later harvested kiwifruit. Antioxidant metabolism has been shown to change with fruit maturity [70], potentially influencing both disorder susceptibility and the interpretation of oxidative stress responses across studies. A simple approach to reduce chilling injuries is to store the fruit at higher temperatures; however, this will often compromise the storage life and so is not acceptable to the industry. Alternative procedures to reduce chilling injuries include pre-storage temperature treatments, which are often referred to as conditioning, or chemicals [12,83,84].

### 6.1. Thermal Treatments

Low-temperature conditioning is achieved by exposing the fruit at a low temperature just above the critical chilling range (the temperature at which chilling injury starts occurring, which for kiwifruit is between 0 and 5 °C, depending upon cultivar [2]), which then results in the fruit having fewer incidences of chilling injury in subsequent low-temperature storage. Temperature conditioning can be achieved in one step or by gradually decreasing the temperature in multiple steps/rates; the latter can be more effective [85]. Low-temperature conditioning in kiwifruit has been shown to inhibit the accumulation of ROS and influence the activities of SOD, CAT, APOX and peroxidase during chilling stress and therefore reduce the incidence of chilling injury [61]. Low-temperature conditioning has also been shown to be effective in other species. Low-temperature conditioning in zucchini (*Cucurbita pepo* L., cv. Elite) increased the activity of APOX and DHAR resulting in fewer chilling injury incidences [86]. Low-temperature conditioning in conjunction with methyl jasmonate treatments increased antioxidant enzyme activity, resulting in the decreased occurrence of chilling injury in bell pepper (*Capsicum annuum* L.) [87] and in peaches (*Prunus persica* Batsch. cv. ‘Baifeng’) [88] and eggplant (*Solanum melongena* L.) [89]. Low-temperature conditioning reduced the incidence of chilling injury and the levels of ROS in mango (*Mangifera indica* L.) [90]. Low-temperature conditioning reduced the peel spot browning that occurs during cold storage in ‘Huangguan’ pears (*Pyrus bretschneideri* Rehd); the low-temperature conditioning resulted in lower activities of PPO and LOX and lower MDA content and maintenance of phenolic compounds [91]. Low-temperature conditioning retained ascorbic acid levels for longer in subsequent cold storage in ‘Star Ruby’ grapefruit (*Citrus paradisi* Macf.) and reduced the incidence of chilling injury [92]. Hot water treatments have also been effective at reducing the incidence of chilling injury in ‘Hongyang’ kiwifruit; the hot water treatment reduced the activity of lipid peroxidase [93]. Intermittent warming occurs during cold storage with periods of warmer temperature exposure; this is effective before chilling injury becomes irreversible. The warmer temperatures allow for normal metabolism and it is thought that repair to any damage that has occurred to membranes, organelles or metabolic pathways from the low temperature can occur, allowing for the removal of toxins or excess intermediates that have occurred [66,85,94,95].

### 6.2. Chemical Treatments

Polyamines also function as antioxidants and play a role in maintaining cellular homeostasis by scavenging ROS. When the levels of polyamines are low, the antioxidant capacity of the fruit is reduced. Additionally, the breakdown of polyamines through polyamine oxidases results in the formation of H_2_O_2_ [96]; this therefore leads to an accumulation of ROS, which can cause oxidative stress, damaging cellular components, accelerating the breakdown of cell structures, and disrupting metabolic processes, thereby promoting senescence. Therefore, the balance of polyamine anabolism and catabolism is critical in managing oxidative stress and delaying senescence in kiwifruit [97]. Polyamines are a group of organic compounds present in all cells; they have a stabilizing effect on membranes and antioxidant activity. Polyamines have also shown to be mediated by ripening and temperature in tomato fruit [98]. Exogenous applications of polyamines have increased the polyamine content in kiwifruit and reduced the incidence of chilling injury [33].

Melatonin can be considered a plant hormone; it interacts with other plant hormones such as ethylene and ABA and is involved in growth, development, fruit ripening, light signal transduction and stress responses in plants [29,99]. Melatonin follows a circadian rhythm, having the highest levels in the night and experiencing decreases in the light [100]. Melatonin also has antioxidant activity, reacting directly with H_2_O_2_, OH^−^, nitric oxide, peroxynitrite and hypochlorous acid, and can also increase/maintain the antioxidant enzyme activities of SOD, CAT, GPOX and GR and decrease the activity of nitric oxide synthase [101,102]. In kiwifruit, melatonin treatment has been shown to reduce chilling injury incidence, increase the activity of antioxidant enzymes (SOD, CAT and APOX), increase levels of ascorbate and glutathione and reduce ROS levels [34]. Melatonin treatments have also been shown to increase the activities of peroxidases, SOD and CAT, reducing H_2_O_2_ and resulting in less membrane damage and delayed dark-induced senescence in leaves [100] and cut flowers [103].

Treatments with methyl jasmonates for kiwifruit have been shown to delay the decrease in antioxidant enzyme activity, resulting in fruit with a longer storage life [104]. In kiwifruit, methyl jasmonate has been shown to reduce chilling injury incidence, increased firmness, decreased respiration, and increased antioxidant enzyme activity (CAT), APOX) [105]. Similar trends have been found in other fruit species. The application of low concentrations (0.01 mM) of methyl jasmonate or methyl salicylate to tomato fruit has been shown to enhance resistance to chilling injuries, and the suggestion is that methyl jasmonate decreases the activity of CAT and therefore the levels of ROS increase. The increase in ROS results in the upregulation of defence-associated pathway genes and a subsequent increase in CAT production and therefore CAT activity, resulting in a heightened ability to respond to the increased levels of ROS that will be produced during storage at low temperatures [106,107]. Treatment with methyl jasmonate for cucumbers and zucchini also reduces the onset of chilling injuries, which is thought to be due to increased levels of polyamines and ABA [43].

Additionally, salicylic acid treatments have been shown to reduce chilling injury in ‘Hayward’ kiwifruit, increasing phenolic compounds [105] and increase in CAT activity and a reduction in lipid peroxidation [108]. In kiwifruit, applications of 1 µmol L^−1^ nitric oxide (NO) have been shown to reduce the accumulation of ROS, increase the activity of SOD and CAT and reduce the activity of peroxidases and lipoxygenases. However, in NO-treated fruit, although ethylene production was delayed, it became higher in treated fruit compared to control fruit after 70 days in cold storage [109]. Treatment with Phytosulfokine-α has been shown to reduce chilling injury in ‘Cuixiang’ kiwifruit, reducing ROS (O_2_^·−^ and H_2_O_2_) and increasing antioxidant enzyme activity (SOD, CAT, APOX and GR) [110]. Treatments with neomycin (inositol 1,4,5-trisphosphat (IP3) inhibitor) and sodium nitroprusside (nitric oxide donor) resulted in reduced chilling injury and reduced lipoxygenase activity in ‘Yate’ kiwifruit [111]. Treatment with γ-Aminobutyric acid (GABA) has been shown to reduce chilling injury incidence and increase ascorbate in ‘Hongyang’ kiwifruit [112].

**Table 2:** Thermal and chemical treatments that have reduced chilling injury incidence in kiwifruit (*Actinidia* sp.) and induced changes in antioxidant metabolism.

Cultivar	Soluble Solid Content at Harvest (%)	Treatment	Time in Storage (Days)	Outcome	Reference
‘Hongyang’	7	Putrescine (2 mM).	60–90	Reduced chilling injury incidence, delayed softening, reduced and delayed ethylene production, inhibited increase in ROS (O_2_^·−^ and H_2_O_2_), increased activity of antioxidant enzymes (SOD and CAT, APOX, GR and DHAR), increased ascorbate and glutathione.	[33]
‘Huayou’	6.5–7.5	Melatonin (0.1 mmol/L).	100	Reduced chilling injury incidence, reduced lignification, decreased electrolyte leakage (MDA), reduced ROS (O_2_^·−^ and H_2_O_2_), increased ascorbate and glutathione levels, increased antioxidant enzyme activity (SOD, CAT and APOX).	[34]
‘Hayward’	7	Low-temperature conditioning (12 °C for 3 days before storage at 0 °C).	120	Reduced chilling injury incidence, lower respiration rate, increased fruit firmness, inhibited membrane permeability, lower ROS production (O_2_^·−^ and H_2_O_2_), increased antioxidant enzyme activity (SOD, peroxidase), reduced antioxidant enzyme activity (CAT, APOX).	[61]
‘Hongyang’	6.95	Hot water treatment (45 °C).	90	Reduced chilling injury incidence, delayed softening, increased SSC, reduced LPOX activity, reduced ethylene.	[93]
‘Hayward’	7.0–7.5	Salicylic acid (1 mM).	126	Reduced chilling injury, delayed softening, reduced respiration, increased phenolics, increased Phenylalanine Ammonia-Lyase activity.	[105]
‘Hayward’	7.0–7.5	Salicylic acid (1 mM).	126	Reduced chilling injury, reduced lipid peroxidase activity, increased CAT activity.	[108]
‘Cuixiang’	6	Phytosulfokine-α (≥95%).	60	Reduced chilling injury, delayed softening, reduced ROS (O_2_^·−^ and H_2_O_2_), increased antioxidant enzyme activity (SOD, CAT, APOX and GR).	[110]
‘Yate’	7	Neomycin or sodium nitroprusside.	40	Reduced chilling injury, reduced lipoxygenase activity.	[111]
‘Hongyang’	7.2	γ-Aminobutyric acid.	100	Reduced chilling injury incidence, increased ascorbate.	[112]
’Xuxiang’	8.0–8.5	Methyl jasmonate (10 μM).	126	Reduced chilling injury incidence, delayed softening, decreased respiration, increased antioxidant enzyme activity (CAT, APOX).	[113]

Note: Soluble solids are used as an indicator of maturity.

Omics approaches, combined with detailed biochemical analyses, are very useful in helping us understand disorders in fruit. For example, RNA-seq studies have been used to investigate the metabolic changes and ROS-related mechanisms associated with the development of chilling injury in kiwifruit and to investigate the mechanisms of treatments that are known to reduce the incidence of chilling injury. For example, salicylic acid was shown to reduce chilling injury incidence and influence genes associated with phenolic metabolism, gibberellins, jasmonic acid and ABA in kiwifruit [105]. Salicylic acid was also shown to influence the expression of genes associated with lipid oxidation, ethylene biosynthesis, cell wall degradation, plant hormone signal transduction and transcription factors, with a mechanistic model for chilling injury mitigation by salicylic treatments developed [108]. In addition, quantitative RT-PCR has been used to investigate the expression levels of genes encoding antioxidant enzyme and phenolic metabolism in kiwifruit treated with melatonin and these genes were upregulated in response to melatonin treatment [34].

RNA-seq studies focusing on chilling injury in kiwifruit have also identified biomarkers which could possibly predict fruit quality after long-term cold storage. The overlying goal was to identify biomarkers that are able to categorize batches of fruit that will develop chilling injury during long-term storage, and therefore a batch of fruit that could be sent to market earlier than those that identified as being suitable for long-term storage [114]. However, the identification of such biomarkers is often simply descriptive, rather than aiming to understand the physiological processes involved in chilling injury in kiwifruit.

## 7. A Model for Showing the Potential Roles of ROS and Antioxidants in the Development of Chilling Injury in Kiwifruit

**Figure 3:**
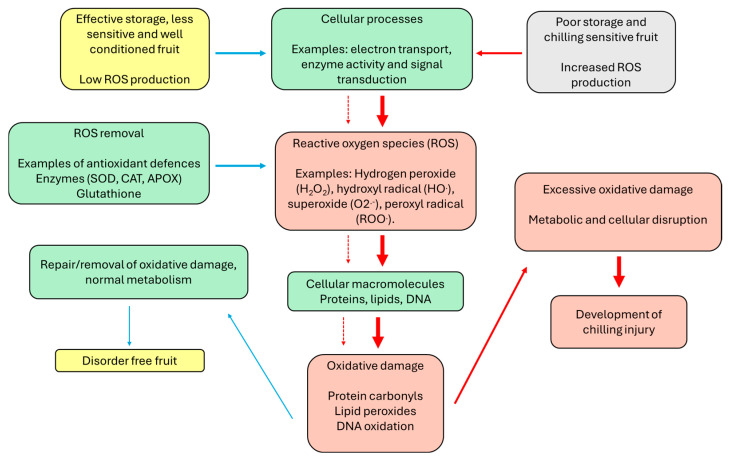
A simple physiological model showing how oxidative damage and antioxidant metabolism could be related to chilling injury in kiwifruit. Blue arrows indicate a positive contribution to oxidative damage mitigation and red arrows indicate a negative contribution to oxidative damage mitigation. Arrows with dotted lines indicate low levels or ROS from well stored fruit. Bold arrows with solid lines indicate higher ROS levels from chilling sensitive and poorly stored fruit.

Based upon our current understanding of the roles of ROS and antioxidant metabolism in cold-stored kiwifruit, the following model for the development of chilling injury in kiwifruit is proposed (Figure 3). Properly conditioned and stored fruit (i.e., harvested at the appropriate maturity, cooled at appropriate rates, stored at suitable temperatures, etc.) will maintain low ROS production and ROS tissue levels, remaining free from chilling injury. Susceptible fruit, e.g., less mature fruit, those cooled too fast and stored temperatures that are not optimal for the cultivar in question, generate higher ROS levels, due to excessive stress-related ROS production or limited ROS scavenging capacity compared to well-conditioned/less susceptible fruit, and develop chilling injury.

However, further in-depth studies on changes in antioxidant metabolism and changes in ROS levels, throughout kiwifruit development and maturation and over several seasons, are required to confirm the above model. In addition, it remains unclear which antioxidants play the most important roles in chilling injury in kiwifruit and if lower antioxidant levels are the primary cause or a consequence of chilling injury. Whether these antioxidant deficiencies occur within particular organelles or throughout entire cells/tissues within kiwifruit is also not yet clear. For instance, it is not yet known whether chromoplast metabolism contributes substantially to ROS production, or whether other cellular compartments/organelles play more critical roles.

Additionally, many studies comparing antioxidant differences among kiwifruit cultivars focus on antioxidants in the context of human nutrition, rather than on their roles within the plant’s responses to stress [115,116,117]. More detailed characterization of cultivar-specific antioxidant metabolism, such as baseline antioxidant levels and the capacity to respond to stress, would provide insight into which aspects of antioxidant regulation contribute to improved storability and reduced incidence of chilling injury.

## 8. Conclusions

Antioxidant metabolism is both a key element and leveraging point in managing the postharvest potential of fruit, including kiwifruit. By integrating preharvest orchard practices and postharvest physiology, future work can better predict, prevent, and manage storage disorders, ultimately improving fruit quality and reducing waste across the supply chain. A better understanding of how oxidative stress and antioxidant pathways influence storage outcomes has the potential to extend shelf life and maintain fruit quality. The susceptibility of kiwifruit to postharvest physiological disorders such as chilling injury is not only determined during storage but is influenced by preharvest conditions. Stress induced in the orchard (abiotic, biotic, or induced by management practices) can influence fruit metabolism, alter the antioxidant balance, and influence how the fruit responds to the additional stress of cold storage. These stress events may occur weeks or months before harvest, even as early as flowering [72], highlighting the complexity of linking environmental cues and orchard practices to storage outcomes.

To fully understand the relationship between stress exposure during fruit development and subsequent development of postharvest disorders, extensive monitoring of oxidative stress markers and antioxidant capacity throughout fruit development and storage is important. Because stress responses are dynamic processes aimed at maintaining homeostasis, single time-point measurements may not capture when and why antioxidant systems fail to protect against damage from ROS. Repeated measurements across storage can more precisely reveal the points at which oxidative balance breaks down, leading to disorder development [32]. This approach could explain why fruit of the same species, or even from the same orchard, differ in susceptibility to chilling injury and other storage disorders. Integrating omics-based approaches (e.g., transcriptomics) with physiological and biochemical measurements can further our understanding of how antioxidant systems interact with other metabolic pathways during fruit development and subsequent low-temperature storage.

For the kiwifruit industry, linking orchard management practices (such as spray timing, canopy management, or water availability) to fruit antioxidant metabolism at harvest may identify strategies that reduce postharvest disorder risk. Breeding or selecting cultivars with more resilient antioxidant metabolism could also be used to reduce the development of postharvest disorders such as chilling injury. Scalable and cost-effective technologies are needed for monitoring oxidative stress and antioxidant capacity in commercial settings. It is also important to note that any new treatments or diagnostic methods must be feasible at the industry scale, be compatible with existing workflows, and be economically viable [118]. From an industry perspective, it is important to distinguish between antioxidant-based strategies that are closest to commercial adoption and those that remain primarily research tools. Approaches such as optimized orchard management, cultivar selection, and postharvest practices that modify oxidative stress are more readily transferable than treatments requiring novel chemistries or specialized infrastructure. However, implementation will ultimately be constrained by cost, regulatory approval, residue considerations, and operational complexity within existing packhouse and storage workflows. Realistically, antioxidant-based strategies are most likely to be adopted where they can be integrated into current kiwifruit cold-chain systems with minimal disruption, low additional cost, and clear benefits to storage performance and disorder reduction. With new genetic tools, it will also be possible to increase the levels of antioxidants in new cultivars.

More information on differences in antioxidant metabolism between commercial varieties of kiwifruit (e.g., ‘Hayward’ and ‘Zesy002’) and on how genetics can contribute to differences in chilling injury susceptibility is also critical. Linked to this, a better understanding of oxidative stress in kiwifruit and the molecular mechanisms associated with the regulation of antioxidant metabolism, including which enzymes are ROS-regulated during kiwifruit ripening and in fruit that do or do not develop chilling injury, would offer clearer mechanistic insights into chilling-injury sensitivity and development in cold stored kiwifruit, helping with the development of more effective mitigation strategies.

## Figures and Tables

**Table 1:** Reactive oxygen species (ROS), mechanisms of production and sites of production in plant cells. Modified from [36].

Site of ROS Production	ROS Type	Mechanism of ROS Production *
Chloroplast	O^·^_2_^−^	ETC
	H_2_O_2_	SOD
	OH	FRs
Mitochondria	O^·^_2_^−^	Respiratory chain
	H_2_O_2_	SOD
	OH	FRs
Cytosol	O^·^_2_^−^	Dehydrogenases and oxidases
	H_2_O_2_	SOD
Peroxisomes	O^·^_2_^−^	ETC
	H_2_O_2_	SOD, oxidases
	OH	FRs
Apoplast	O^·^_2_^−^	NAPH oxidases
	H_2_O_2_	SOD
	OH	FRs, Peroxidases

* Electron transport chain (ETC) refers to electrons that are transported across the electron transport chain. Superoxide dismutase (SOD). Fenton reactions (FRs).

## Data Availability

No new data were created or analyzed in this study.

## Abbreviations

APOX	Ascorbate peroxidase
CAT	Catalase
CI	Chilling injury
DHAR	Dehydroascorbate peroxidase
GPOX	Glutathione peroxidase
GR	Glutathione reductase
MDHAR	monodhydroascrobate peroxidase
PC	Protein carbonyl
ROS	Reactive oxygen species
SOD	Superoxide dismutase

## References

[1] Ferguson A.R. (2023). Biology of the Genus *Actinidia*. Kiwifruit: Botany, Production and Uses.

[2] Burdon J., Lallu N., Yahia E.M. (2011). 14—Kiwifruit (*Actinidia* spp.). Postharvest Biology and Technology of Tropical and Subtropical Fruits.

[3] Ferguson A.R., Huang H., Costa G. (2023). History of Kiwifruit: Evolution of a Global Crop.

[4] Ferguson A.R., Seal A. (2008). Kiwifruit. Temperate Fruit Crop Breeding: Germplasm to Genomics.

[5] Obadovic I., Burdon J. Impact case study of maturity research for the New Zealand kiwifruit industry. Proceedings of the XI International Symposium on Kiwifruit.

[6] Beever D.J., Hopkirk G. (1990). Fruit development and fruit physiology. Kiwifruit: Science and Management.

[7] Bano S., Scrimgeour F. (2012). The export growth and revealed comparative advantage of the New Zealand kiwifruit industry. Int. Bus. Res..

[8] Wang R., Martin P., McAtee P.A., Schaffer R.J., Burdon J. (2022). New insights into the storage performance of ‘Hayward’ kiwifruit from a comparison of three cultivars. N. Z. J. Crop Hortic. Sci..

[9] Jayas D.S., Jeyamkondan S. (2002). Postharvest Technology: Modified Atmosphere Storage of Grains Meats Fruits and Vegetables. Biosyst. Eng..

[10] Kader A.A. (2002). Postharvest Technology of Horticultural Crops.

[11] Ekman J.H., Golding J., McGlasson W. (2005). Innovation in cold storage technologies. Stewart Postharvest Rev..

[12] Wang C.Y. (1993). Approaches to Reduce Chilling Injury of Fruits and Vegetables. Horticultural Reviews.

[13] Biswas P., Brummell D.A., de Freitas S.T., Pareek S. (2019). Chilling injury. Postharvest Physiological Disorders in Fruits and Vegetables.

[14] Burdon J., Wang R. (2023). Postharvest: Fresh Fruit Harvest, Storage and Supply.

[15] Burdon J.N. (2018). Kiwifruit biology: The commercial implications of fruit maturation. Hortic. Rev..

[16] Feng J., Maguire K.M., MacKay B.R. (2006). Discriminating batches of ‘Hayward’ kiwifruit for storage potential. Postharvest Biol. Technol..

[17] Decros G., Baldet P., Beauvoit B., Stevens R., Flandin A., Colombié S., Gibon Y., Pétriacq P. (2019). Get the Balance Right: ROS Homeostasis and Redox Signalling in Fruit. Front. Plant Sci..

[18] Nardozza S., Burdon J.N. (2023). Fruit development: Growth, maturation and ripening. Kiwifruit: Botany, Production and Uses.

[19] Ishangulyyev R., Kim S., Lee S. (2019). Understanding Food Loss and Waste—Why Are We Losing and Wasting Food?. Foods.

[20] Yang Q., Rao J., Yi S., Meng K., Wu J., Hou Y. (2012). Antioxidant enzyme activity and chilling injury during low-temperature storage of Kiwifruit cv. Hongyang exposed to gradual postharvest cooling. Hortic. Environ. Biotechnol..

[21] Gong D., Bi Y., Li Y., Wang Y., Prusky D., Alkan N. (2022). Preharvest elicitors spray improves antioxidant activity, alleviates chilling injury, and maintains quality in harvested fruit. Horticulturae.

[22] Apel K., Hirt H. (2004). Reactive Oxygen Species: Metabolism, Oxidative Stress, and Signal Transduction. Annu. Rev. Plant Biol..

[23] Hasanuzzaman M., Bhuyan M.H.M.B., Parvin K., Bhuiyan T.F., Anee T.I., Nahar K., Hossen M.S., Zulfiqar F., Alam M.M., Fujita M. (2020). Regulation of ROS Metabolism in Plants under Environmental Stress: A Review of Recent Experimental Evidence. Int. J. Mol. Sci..

[24] Foyer C.H., Noctor G. (2003). Redox sensing and signalling associated with reactive oxygen in chloroplasts, peroxisomes and mitochondria. Physiol. Plant..

[25] Foyer C.H., Noctor G. (2005). Redox Homeostasis and Antioxidant Signaling: A Metabolic Interface between Stress Perception and Physiological Responses. Plant Cell.

[26] Meitha K., Pramesti Y., Suhandono S. (2020). Reactive Oxygen Species and Antioxidants in Postharvest Vegetables and Fruits. Int. J. Food Sci..

[27] Huan C., Jiang L., An X., Yu M., Xu Y., Ma R., Yu Z. (2016). Potential role of reactive oxygen species and antioxidant genes in the regulation of peach fruit development and ripening. Plant Physiol. Biochem..

[28] Miller G., Shulaev V., Mittler R. (2008). Reactive oxygen signaling and abiotic stress. Physiol. Plant.

[29] Cao S., Shao J., Shi L., Xu L., Shen Z., Chen W., Yang Z. (2018). Melatonin increases chilling tolerance in postharvest peach fruit by alleviating oxidative damage. Sci. Rep..

[30] Biswas P., East A., Hewett E., Heyes J. (2017). Chilling Injury in Tomato Fruit. Hortic. Rev..

[31] Albornoz K., Zhou J., Yu J., Beckles D.M. (2022). Dissecting postharvest chilling injury through biotechnology. Curr. Opin. Biotechnol..

[32] Toivonen P.M.A. (2004). Postharvest Storage Procedures and Oxidative Stress. HortScience.

[33] Yang Q., Wang F., Rao J. (2016). Effect of Putrescine Treatment on Chilling Injury, Fatty Acid Composition and Antioxidant System in Kiwifruit. PLoS ONE.

[34] Jiao J., Jin M., Liu H., Suo J., Yin X., Zhu Q., Rao J. (2022). Application of melatonin in kiwifruit (*Actinidia chinensis*) alleviated chilling injury during cold storage. Sci. Hortic..

[35] Gill S.S., Tuteja N. (2010). Reactive oxygen species and antioxidant machinery in abiotic stress tolerance in crop plants. Plant Physiol. Biochem..

[36] Jajic I., Sarna T., Strzalka K. (2015). Senescence, Stress, and Reactive Oxygen Species. Plants.

[37] Jaspers P., Kangasjärvi J. (2010). Reactive oxygen species in abiotic stress signaling. Physiol. Plant.

[38] Wei W., Liu Z., Pan X., Yang T., An C., Wang Y., Li L., Liao W., Wang C. (2025). Effects of reactive oxygen species on fruit ripening and postharvest fruit quality. Plant Sci..

[39] Schieber M., Chandel N.S. (2014). ROS Function in Redox Signaling and Oxidative Stress. Curr. Biol..

[40] Nyström T. (2005). Role of oxidative carbonylation in protein quality control and senescence. EMBO J..

[41] Gutteridge J.M. (1995). Lipid peroxidation and antioxidants as biomarkers of tissue damage. Clin. Chem..

[42] Anjum N.A., Sofo A., Scopa A., Roychoudhury A., Gill S.S., Iqbal M., Lukatkin A.S., Pereira E., Duarte A.C., Ahmad I. (2015). Lipids and proteins—Major targets of oxidative modifications in abiotic stressed plants. Environ. Sci. Pollut. Res..

[43] Bartz J.A., Brecht J.K. (2002). Postharvest Physiology and Pathology of Vegetables.

[44] Saltveit M.E. (2002). The rate of ion leakage from chilling-sensitive tissue does not immediately increase upon exposure to chilling temperatures. Postharvest Biol. Technol..

[45] Mittler R., Zandalinas S.I., Fichman Y., Van Breusegem F. (2022). Reactive oxygen species signalling in plant stress responses. Nat. Rev. Mol. Cell Biol..

[46] Foyer C.H., Noctor G. (2011). Ascorbate and Glutathione: The Heart of the Redox Hub. Plant Physiol..

[47] Perotti V.E., Moreno A.S., Podestá F.E. (2014). Physiological aspects of fruit ripening: The mitochondrial connection. Mitochondrion.

[48] Li L., Yuan H. (2013). Chromoplast biogenesis and carotenoid accumulation. Arch. Biochem. Biophys..

[49] Zhang B., Yin X.-r., Li X., Yang S.-l., Ferguson I.B., Chen K.-s. (2009). Lipoxygenase Gene Expression in Ripening Kiwifruit in Relation to Ethylene and Aroma Production. J. Agric. Food Chem..

[50] Antunes M. (2007). The role of ethylene in kiwifruit ripening and senescence. Stewart Postharvest Rev..

[51] Kim H., Hewett E., Lallu N. The role of ethylene in kiwifruit softening. Proceedings of the IV International Symposium on Kiwifruit.

[52] Feng J., Maguire K.M., MacKay B.R. (2003). Factors Affecting Ethylene Production of Hayward Kiwifruit. Acta Hortic..

[53] Günther C.S., Marsh K.B., Winz R.A., Harker R.F., Wohlers M.W., White A., Goddard M.R. (2015). The impact of cold storage and ethylene on volatile ester production and aroma perception in ‘Hort16A’ kiwifruit. Food Chem..

[54] Figueroa C.R., Jiang C.-Z., Torres C.A., Fortes A.M., Alkan N. (2021). Regulation of fruit ripening and senescence. Front. Plant Sci..

[55] Woo H.R., Masclaux-Daubresse C., Lim P.O. (2018). Plant senescence: How plants know when and how to die. J. Exp. Bot..

[56] Hussain A., Shah F., Ali F., Yun B.-W. (2022). Role of Nitric Oxide in Plant Senescence. Front. Plant Sci..

[57] Xia Y., Chen T., Qin G., Li B., Tian S. (2016). Synergistic action of antioxidative systems contributes to the alleviation of senescence in kiwifruit. Postharvest Biol. Technol..

[58] Zhang B., Chen K., Bowen J., Allan A., Espley R., Karunairetnam S., Ferguson I. (2006). Differential expression within the LOX gene family in ripening kiwifruit. J. Exp. Bot..

[59] Wu J., Tang R., Fan K. (2024). Recent advances in postharvest technologies for reducing chilling injury symptoms of fruits and vegetables: A review. Food Chem. X.

[60] Franzoni G., Spadafora N.D., Sirangelo T.M., Ferrante A., Rogers H.J. (2023). Biochemical and molecular changes in peach fruit exposed to cold stress conditions. Mol. Hortic..

[61] Yang Q., Zhang Z., Rao J., Wang Y., Sun Z., Ma Q., Dong X. (2013). Low-temperature conditioning induces chilling tolerance in ‘Hayward’ kiwifruit by enhancing antioxidant enzyme activity and regulating en-dogenous hormones levels. J. Sci. Food Agric..

[62] Li Z., Wang L., Xie B., Hu S., Zheng Y., Jin P. (2020). Effects of exogenous calcium and calcium chelant on cold tolerance of postharvest loquat fruit. Sci. Hortic..

[63] Wongs-Aree C., Aschariyaphotha W., Palapol Y., Bodhipadma K., Noichinda S. (2024). Structural membrane alterations in tropical horticultural crops under postharvest chilling stress. Veg. Res..

[64] Lallu N. (1997). Low Temperature Breakdown in Kiwifruit. Acta Hortic..

[65] Gwanpua S.G., Saeed M., Jabbar A., Heyes J. (2019). Kiwifruit. Postharvest Physiological Disorders in Fruits and Vegetables.

[66] Wang C.Y. (1994). Chilling Injury of Tropical Horticultural Commodities. HortScience.

[67] Lurie S., Crisosto C.H. (2005). Chilling injury in peach and nectarine. Postharvest Biol. Technol..

[68] Alavi M., Fullerton C.G., Pidakala P., Burdon J.N. (2022). Prediction of chilling injury risk in ‘Zesy002’kiwifruit from softening early in storage. N. Z. J. Crop Hortic. Sci..

[69] Burdon J., Pidakala P., Martin P., Billing D., Boldingh H. (2016). Fruit maturation and the soluble solids harvest index for ‘Hayward’ kiwifruit. Sci. Hortic..

[70] Wang F., Yang Q., Zhao Q., Zhang X. (2020). Roles of antioxidant capacity and energy metabolism in the maturity-dependent chilling tolerance of postharvest kiwifruit. Postharvest Biol. Technol..

[71] Hodges D.M., DeLong J.M. (2007). The relationship between antioxidants and postharvest storage quality of fruits and vegetables. Stewart Postharvest Rev..

[72] Hodges D., Lester G., Pennell K., Toivonen P. (2004). Oxidative Stress: Importance for Postharvest Quality. Hortscience.

[73] Wang X., Wei Y., Jiang S., Ye J., Chen Y., Xu F., Shao X. (2024). Transcriptome analysis reveals that trehalose alleviates chilling injury of peach fruit by regulating ROS signaling pathway and enhancing antioxidant capacity. Food Res. Int..

[74] Einset J., Per W., Bones A. (2007). ROS Signaling Pathways in Chilling Stress. Plant Signal. Behav..

[75] Yadav M.R., Choudhary M., Singh J., Lal M.K., Jha P.K., Udawat P., Gupta N.K., Rajput V.D., Garg N.K., Maheshwari C. (2022). Impacts, tolerance, adaptation, and mitigation of heat stress on wheat under changing climates. Int. J. Mol. Sci..

[76] Kapoor D., Sharma R., Handa N., Kaur H., Rattan A., Yadav P., Gautam V., Kaur R., Bhardwaj R. (2015). Redox homeostasis in plants under abiotic stress: Role of electron carriers, energy metabolism mediators and proteinaceous thiols. Front. Environ. Sci..

[77] Jogawat A., Yadav B., Chhaya, Lakra N., Singh A.K., Narayan O.P. (2021). Crosstalk between phytohormones and secondary metabolites in the drought stress tolerance of crop plants: A review. Physiol. Plant..

[78] Belay Z.A., James Caleb O. (2022). Role of integrated omics in unravelling fruit stress and defence responses during postharvest: A review. Food Chem. Mol. Sci..

[79] Goulao L., Oliveira C. (2008). Cell wall modifications during fruit ripening: When a fruit is not the fruit. Trends Food Sci. Technol..

[80] Le Gall H., Philippe F., Domon J.-M., Gillet F., Pelloux J., Rayon C. (2015). Cell Wall Metabolism in Response to Abiotic Stress. Plants.

[81] Wang L., Chen S., Shao J., Zhang C., Mei L., Wang K., Jin P., Zheng Y. (2022). Hydrogen sulfide alleviates chilling injury in peach fruit by maintaining cell structure integrity via regulating endogenous H2S, antioxidant and cell wall metabolisms. Food Chem..

[82] Yao M., Ge W., Zhou Q., Zhou X., Luo M., Zhao Y., Wei B., Ji S. (2021). Exogenous glutathione alleviates chilling injury in postharvest bell pepper by modulating the ascorbate-glutathione (AsA-GSH) cycle. Food Chem..

[83] Burdon J., Lallu N., Haynes G., Pidakala P., Billing D., McDermott K. (2010). Dynamic Controlled Atmosphere Storage of New Zealand-Grown ‘Hass’ Avocado Fruit. Acta Hortic..

[84] Lallu N., Burdon J., Yearsley C.W., Billing D. (2003). Commercial Practices Used for Controlled Atmosphere Storage of Hayward Kiwifruit.

[85] Patel B., Tandel Y., Patel A., Patel B. (2016). Chilling Injury in Tropical and Subtropical Fruits: A Cold Storage Problem and Its Remedies: A Review. Int. J. Sci. Environ. Technol..

[86] Wang C.Y. (1996). Temperature preconditioning affects ascorbate antioxidant system in chilled zucchini squash. Postharvest Biol. Technol..

[87] Wang Y., Gao L., Wang Q., Zuo J. (2019). Low temperature conditioning combined with methyl jasmonate can reduce chilling injury in bell pepper. Sci. Hortic..

[88] Jin P., Wang K., Shang H., Tong J., Zheng Y. (2009). Low-temperature conditioning combined with methyl jasmonate treatment reduces chilling injury of peach fruit. J. Sci. Food Agric..

[89] Shi J., Zuo J., Xu D., Gao L., Wang Q. (2019). Effect of low-temperature conditioning combined with methyl jasmonate treatment on the chilling resistance of eggplant (*Solanum melongena* L.) fruit. J. Food Sci. Technol..

[90] Zhang Z., Zhu Q., Hu M., Gao Z., An F., Li M., Jiang Y. (2017). Low-temperature conditioning induces chilling tolerance in stored mango fruit. Food Chem..

[91] Li D., Cheng Y., Dong Y., Shang Z., Guan J. (2017). Effects of low temperature conditioning on fruit quality and peel browning spot in ‘Huangguan’ pears during cold storage. Postharvest Biol. Technol..

[92] Chaudhary P.R., Jayaprakasha G.K., Porat R., Patil B.S. (2014). Low temperature conditioning reduces chilling injury while maintaining quality and certain bioactive compounds of ‘Star Ruby’ grapefruit. Food Chem..

[93] Ma Q., Suo J., Huber D.J., Dong X., Han Y., Zhang Z., Rao J. (2014). Effect of hot water treatments on chilling injury and expression of a new C-repeat binding factor (CBF) in ‘Hongyang’kiwifruit during low temperature storage. Postharvest Biol. Technol..

[94] Gwanpua S.G., Jabbar A., Zhao M., Heyes J.A., East A.R. (2018). Investigating the potential of dual temperature storage as a postharvest management practice to mitigate chilling injury in kiwifruit. Int. J. Refrig..

[95] Biswas P., East A., Hewett E., Heyes J. (2016). Intermittent warming in alleviating chilling injury—A potential technique with commercial constraint. Food Bioprocess Technol..

[96] Gao F., Mei X., Li Y., Guo J., Shen Y. (2021). Update on the Roles of Polyamines in Fleshy Fruit Ripening, Senescence, and Quality. Front. Plant Sci..

[97] Gupta K., Sengupta A., Chakraborty M., Gupta B. (2016). Hydrogen Peroxide and Polyamines Act as Double Edged Swords in Plant Abiotic Stress Responses. Front. Plant Sci..

[98] Tsaniklidis G., Charova S.N., Fanourakis D., Tsafouros A., Nikoloudakis N., Goumenaki E., Tsantili E., Roussos P.A., Spiliopoulos I.K., Paschalidis K.A. (2021). The role of temperature in mediating postharvest polyamine homeostasis in tomato fruit. Postharvest Biol. Technol..

[99] Arnao M.B., Hernández-Ruiz J. (2020). Melatonin in flowering, fruit set and fruit ripening. Plant Reprod..

[100] Zhang J., Li H., Xu B., Li J., Huang B. (2016). Exogenous Melatonin Suppresses Dark-Induced Leaf Senescence by Activating the Superoxide Dismutase-Catalase Antioxidant Pathway and Down-Regulating Chlorophyll Degradation in Excised Leaves of Perennial Ryegrass (*Lolium perenne* L.). Front. Plant Sci..

[101] Reiter R.J., Tan D.X. (2002). Melatonin: An antioxidant in edible plants. Ann. N. Y. Acad. Sci..

[102] Arnao M.B., Hernández-Ruiz J. (2019). Melatonin: A New Plant Hormone and/or a Plant Master Regulator?. Trends Plant Sci..

[103] Safaei Far A., Mousavi-Fard S., Rezaei Nejad A., Shahbazi F., Ahmadi-Majd M., Fanourakis D. (2024). Nano Silver and melatonin effectively delay the senescence of cut carnation flowers under simulated vibrational stress. J. Hortic. Sci. Biotechnol..

[104] Xie G., Liu N., Zhang Y., Tan S., Xu Y., Luo Z. (2024). Postharvest MeJA maintains the shelf quality of kiwifruit after cold storage by regulating antioxidant capacity and activating the disease resistance. Postharvest Biol. Technol..

[105] Niu Y., Ye L., Wang Y., Shi Y., Liu Y., Luo A. (2023). Transcriptome analysis reveals salicylic acid treatment mitigates chilling injury in kiwifruit by enhancing phenolic synthesis and regulating phytohormone signaling pathways. Postharvest Biol. Technol..

[106] Ding C.-K., Wang C.Y., Gross K.C., Smith D.L. (2001). Reduction of chilling injury and transcript accumulation of heat shock proteins in tomato fruit by methyl jasmonate and methyl salicylate. Plant Sci..

[107] Ding C.-K., Wang C., Gross K.C., Smith D.L. (2002). Jasmonate and salicylate induce the expression of pathogenesis-related-protein genes and increase resistance to chilling injury in tomato fruit. Planta.

[108] Niu Y., Ye L., Wang Y., Shi Y., Luo A. (2024). Salicylic acid mitigates ‘Hayward’ kiwifruit chilling injury by regulating hormone and proline metabolism, as well as maintaining cellular structure. Food Biosci..

[109] Zhu S., Sun L., Liu M., Zhou J. (2008). Effect of nitric oxide on reactive oxygen species and antioxidant enzymes in kiwifruit during storage. J. Sci. Food Agric..

[110] Wang D., Ren X., Meng L., Zheng R., Li D., Kong Q. (2023). Exogenous Phytosulfokine α (PSKα) Alleviates Chilling Injury of Kiwifruit by Regulating Ca^2+^ and Protein Kinase-Mediated Reactive Oxygen Species Metabolism. Foods.

[111] Jiao C. (2021). IP3 mediates NO-enhanced chilling tolerance in postharvest kiwifruit. Postharvest Biol. Technol..

[112] Liu Q., Li X., Jin S., Dong W., Zhang Y., Chen W., Shi L., Cao S., Yang Z. (2023). gamma-Aminobutyric acid treatment induced chilling tolerance in postharvest kiwifruit (*Actinidia chinensis* cv. Hongyang) via regulating ascorbic acid metabolism. Food Chem..

[113] Niu Y., Ye L., Wang Y., Shi Y., Luo A. (2024). Role of methyl jasmonate in alleviating chilling injury in ‘Xuxiang’ kiwifruit: Insights from transcriptomic evidence. Postharvest Biol. Technol..

[114] Favre L., Hunter D.A., O’Donoghue E.M., Erridge Z.A., Napier N.J., Cho J., Nangul A., O’Donnell K., Pidakala P., Martin P. (2023). Maturity biomarkers predicting storage performance of early-harvested yellow-fleshed kiwifruit identified using integrated multi-omics analysis. Postharvest Biol. Technol..

[115] Ozen T., Zenginbal H., Yazicioglu E., Gul F., Demirtas I. (2018). A Comparison Investigation on Antioxidant Activities, Physicochemical Properties and Phytochemical Contents of Kiwifruit Genotypes and Cultivars. Int. J. Fruit Sci..

[116] Ma T., Sun X., Zhao J., You Y., Lei Y., Gao G., Zhan J. (2017). Nutrient compositions and antioxidant capacity of kiwifruit (Actinidia) and their relationship with flesh color and commercial value. Food Chem..

[117] Nishiyama I., Yamashita Y., Yamanaka M., Shimohashi A., Fukuda T., Oota T. (2004). Varietal Difference in Vitamin C Content in the Fruit of Kiwifruit and Other *Actinidia* Species. J. Agric. Food Chem..

[118] Kitinoja L., Saran S., Roy S.K., Kader A.A. (2011). Postharvest technology for developing countries: Challenges and opportunities in research, outreach and advocacy. J. Sci. Food Agric..

